# Design and evaluation of a unique RT-qPCR assay for diagnostic quality control assessment that is applicable to pathogen detection in three species of salmonid fish

**DOI:** 10.1186/1746-6148-9-183

**Published:** 2013-09-16

**Authors:** Dagoberto Sepúlveda, Harry Bohle, Álvaro Labra, Horst Grothusen, Sergio H Marshall

**Affiliations:** 1 Laboratorio de Genética e Inmunología Molecular, Instituto de Biología, Facultad de Ciencias, Pontificia Universidad Católica de Valparaíso, Valparaíso, Chile; 2 Laboratorio de Patógenos Acuícolas, Núcleo Biotecnología Curauma, Pontificia Universidad Católica de Valparaíso, Valparaíso, Chile; 3 ADL Diagnostic Chile Ltda., Laboratorio de Diagnóstico y Biotecnología, Puerto Montt, Chile; 4 Servicio Nacional de Pesca (Sernapesca), Valparaíso, Chile; 5 Fraunhofer Chile Research Foundation, Center For Systems Biotechnology, Santiago, Chile; 6 Present Address: Department Animal Science, Fish Health, Aarhus University, Hangøvej 2, 8200 Aarhus N, Denmark

**Keywords:** Elongation Factor 1 alpha, ELF1α, Endogenous control, Fish disease, Fish virus, Real-time PCR, RT-qPCR

## Abstract

**Background:**

The detection of pathogens at early stages of infection is a key point for disease control in aquaculture. Therefore, accurate diagnostic procedures are a must. Real-time PCR has been a mainstay in diagnostics over the years due to its speed, specificity, sensitivity, reproducibility and throughput; as such, real-time PCR is a target for improvement. Nevertheless, to validate a novel diagnostic tool, correct setup of the assay, including proper endogenous controls to evaluate the quantity and quality of the samples and to detect possible sample degradation, is compulsory. This work aims to design a unique RT-qPCR assay for pathogen detection in the three salmonid species reared in Chile. The assay uses elongation factor 1 alpha as the single endogenous control, thus avoiding the need for multiple endogenous controls, as well as multiple validations and non-comparable quality control parameters.

**Results:**

The *in vivo* and *in vitro* analyses of samples from *Salmo salar*, *Oncorhynchus mykiss* and *Oncorhynchus kisutch* showed that when primers were accurately selected to target conserved regions of the elongation factor 1 alpha (ELF1α) gene, a single novel RT-qPCR assay yielding similar and reproducible Ct values between the three species could be designed. The opposite occurred when an assay originally designed for *Salmo salar* was tested in samples from the two species of the genus *Oncorhynchus*.

**Conclusions:**

Here, we report the design and evaluation of an accurate trans-species RT-qPCR assay that uses the elongation factor 1 alpha (ELF1α) gene as an endogenous control and is applicable for diagnostic purposes in samples obtained from the three salmonid species reared in Chile.

## Background

In nature, fish are exposed to a wide variety of microorganisms of bacterial, viral or fungal origins, many of which are capable of causing disease. When fish are reared under controlled conditions, the menace of disease-causing agents increases significantly because confined fish are also exposed to a number of stressors, such as handling, transport, poor water quality and overstocking. These stressors, together with other weaknesses such as physiological unbalance or nutritional deficiency, challenge homeostasis and thus allow opportunistic infections to proceed [1]. As a result, diseases represent a major economic cost, especially when the impact is on fish of high commercial value [2]. Some of these pathogens are fastidious and highly aggressive, causing high mortality rates, while others persist in fish, representing a potential danger to managed fish in aquaculture if an outburst occurs close to harvest. In this scheme, accurate pathogen detection, ideally in the early stages of infection, is necessary in order to design adequate strategies to control key infectious diseases that seriously threaten the sustainability of aquaculture. One of the techniques that has consistently been transversal in specifically detecting sequences of target pathogens in humans, plants and animals, including fish, is real-time PCR because of its sensitivity, specificity, speed, throughput and reproducibility [3]. In the aquaculture environment, this technique has been successfully used to detect, among other pathogens, viral hemorrhagic septicemia virus (VHSV) [4], infectious hematopoietic necrosis virus (IHNV) [5], salmon alphavirus (SAV) [6], *Piscirickettsia salmonis*[7-9], *Renibacterium salmoninarum*[10]*,* infectious salmon anemia virus (ISAV) [11], and infectious pancreatic necrosis virus (IPNV) [12].

Nevertheless, massive application of this technique requires correct setup of the assay, including the necessary controls, for unequivocal interpretation of the results. Among the most important of these controls is an endogenous reference control that is able to provide information not only about the amount of the detectable target nucleic acid in the sample to be analyzed but also about the sample integrity in order to accurately validate processes such as sampling, transport, and nucleic acid isolation, allowing degradation of the samples to be detected [11,13]. This is not a simple task to achieve, as determining the proper endogenous levels of a given marker molecule depends on a number of variables, including the target organ and the type of nucleic acid to be detected. In the case of targeting viral RNA, specifically for the diagnosis of the ISA virus in infected tissues of fish, the use of the elongation factor 1 alpha (ELF1α) has been demonstrated to be a reliable reference RNA-expressed control because of its stability and constant expression in *Salmo salar* organs, in both the presence and the absence of the ISA virus [11,14-16]. Additionally, because of its relevance, ELF1α has also been recommended as a housekeeping gene for gene expression analyses of other salmonid fish-related pathogens, such as *Piscirickettsia salmonis*[17], SAV [18], and VHSV [4].

Although an ELF1α-driven assay has been successfully used for ISAV detection in *Salmo salar* samples [11,14], it is also known to have a highly variable performance when used for the diagnosis of other reared salmonid species, particularly *Oncorhynchus kisutch* and *Oncorhynchus mykiss,* which together with *Salmo salar*, constitute the economically important species cultivated in Chile. For this reason, we considered it imperative to analyze the sequence variability associated with a presumptive polymorphism in the ELF1α coding sequence of the three salmonid species cultivated in Chile; we would then be able to design a transversal RT-qPCR assay that would allow us to use the same parameters to accurately determine the quantity and quality of the samples, regardless of the salmonid species tested and thereby reliably diagnose RNA pathogens.

## Results and discussion

### Sequence variability evaluation

**Figure 1:**
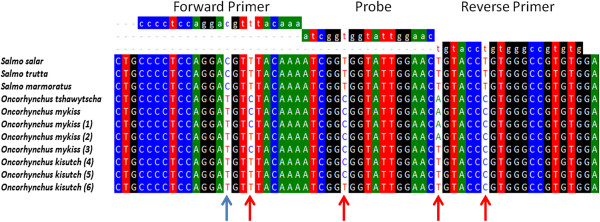
**Analysis of nucleotide variation between different salmonid species.** A multiple alignment was made using ClustalW [23] using the Bioedit software [24]. Top, primers and probe used in the current assay (ELF1α) [14]. The first 5 species sequences are available in the GenBank database; *S. salar* [BT060430], *S. marmoratus* [EU853442], *S. trutta* [EF406271]*, O. tshawytscha* [FJ890356] *and O. mykiss* [AF498320]. Samples labeled with numbers from 1 to 6 were representative of sequences obtained from *Oncorhynchus* samples in this work. Blue arrow shows the Intragenus conserved variation and red arrow show the Intragenus non-conserved variations.

Because salmon production in Chile relies on two additional species that are different from *Salmo salar,* we decided to compare the potential nucleotide variability in the RT-qPCR amplifiable target region of ELF1α [11,14] in all three salmonid fish species reared in Chile, as variability in this region could be causing the species-dependent performance of the assay. cDNAs from 20 tissue samples from *Oncorhynchus mykiss* and *Oncorhynchus kisutch* were amplified, and the PCR products were sequenced directly to avoid any sequence selection bias that might result from a plasmid cloning process. The electropherogram profiles (data not shown) consistently showed double peaks at four positions on the amplified region: one at the forward primer annealing position, one at the probe annealing position, and two at the reverse primer annealing position (Figure 1). It is particularly relevant that one of the two mismatches detected by the reverse primer was situated at the 3′ end, which could seriously interfere with the efficiency of the amplification process [19]. According to previous studies, the presence of double peaks in the electropherograms could be explained by the presence of heterozygosity, SNPs or sequence insertions [20-22]. Although not the main focus of this work, the information obtained here allowed us to design novel transversal primers and probes that are able to specifically amplify and detect selected ELF1α coding sequences in the three fish species reared in Chile with the same degree of accuracy.

To determine whether differences detected in the amplification could be overcome to generate a transversal RT-qPCR reaction, multiple alignments were made with the sequences derived from this study, as well as with those available in the GenBank database. Figure 1 shows that using the BioEdit software [24], it was possible to identify two types of nucleotide variations where we could detect double peaks in the electropherograms. First, an “intragenus conserved variation” consisted of a cytidine residue in the forward primer annealing position for all species of the *Salmo* genus analyzed, which was changed to a Thymidine residue for the two species of the *Oncorhynchus* genus analyzed (blue arrow in Figure 1). Additionally, we identified an “intragenus non-conserved variation,” which did not correlate with specific changes in each genus (red arrow in Figure 1).

### Design of a new transversal assay

Considering the nucleotide variations previously shown, we were in a position to design a new assay to detect ELF1α in all salmonid fish species cultivated in Chile. It is well-known that nucleotide variations in the annealing positions of primers and probes affect the sensitivity and efficiency of this type of reaction, as they can lead to putative underestimations of the actual amount of template present in a sample. Although it may seem trivial, mismatches between primers and probes on a given template actually constitutes an important issue to consider in the generation of a highly specific and reproducible detection assay [19,25]. We chose to use degenerate nucleotides in the probes and primers involving internal nucleotide variations as a testing strategy. Additionally, we moved the reverse primer one nucleotide downstream from the probe, taking into account that a variation in the 3′ end could dramatically affect the performance of the assay. Table 1 shows the three assay conditions evaluated and compared in this work. Assay N°1 (ELF1α) is the assay currently in use [14]. Assay N°2 (ELF1α GIM-1), uses the same sequences as assay N°1, but incorporates degeneracy in the variable positions. Assay N°3 (ELF1α GIM-2) employs different primers to eliminate the 3′ end degeneration in the original reverse primer. The same probe was used for assays N°2 and N°3, which differ from each other only in terms of the primers used.

**Table 1:** Alternative assays used to compare efficiency and transversality of detection

**Assay N°**	**Assay name**	**Name**	**Sequence (5′ - 3′)**	**Reference**
1		ELF1α For	CCCCTCCAGGACGTTTACAAA	[14]
1	ELF1α	ELF1α Rev	CACACGGCCCACAGGTACA	[14]
1		FAM-ELF1α	FAM- ATCGGCGGTATTGGAAC	[14]
2		ELF1α GIM-1-For	CCCCTCCAGGAYGTYTACAAA	This work
2	ELF1α GIM-1	ELF1α GIM-1 Rev	CACACGGCCCACRGGTACW	This work
2		FAM- ELF1α GIM	FAM-ATCGGYGGTAT + T + G + G + A + AC-BHQ	This work
3		ELF1α GIM-2 For	GCCCCTCCAGGAYGTYTACAA	This work
3	ELF1α GIM-2	ELF α GIM-2 Rev	CCACACGGCCCACRGGTAC	This work
3		FAM-ELF1α GIM	FAM-ATCGGYGGTAT + T + G + G + A + AC-BHQ	This work

Assay N°1 (ELF1α) is the current assay. Assays N°2 (ELF1α GIM-1) and N°3 (ELF1α GIM-2), use alternative primers and probes designed to test the objectives of this study. The FAM-ELF1α probe has MGB, and the FAM-ELF1α-GIM probe has LNA residues, denoted by a + symbol before the corresponding nucleotide.

### Evaluation of the alternative new assays

Initially, performances of the two novel alternative assays (N°2 and N°3) were compared against the classical one (N°1) using templates obtained from *S. salar*, *O. kisutch* and *O. mykiss* tissue samples. For this comparison, the detecting probe was used without an MGB or LNA system [13]. Under these conditions, the probe displayed a Tm value of 49.9°C, which was calculated using OligoCalc [26] and which suggested low stability in the annealing process of the probe and template. This allowed us to evaluate the performance of the assay based on the condition that any mismatch could affect the results considerably, thus making it easier to detect any improvement.

The results of the three assays in Table 2 clearly show that assay N°3 performed better than assays N°1 and N°2 and was therefore selected for further evaluation. Assay N°3 had lower Ct values and higher relative fluorescence than the other two assays for all samples evaluated. As expected, the Ct value differences between assays N°1 and N°3 were higher between samples from *O. mykiss* and *O. kisutch* species. This is because assay N°3 takes into account the nucleotide variability in the amplification region for this genus, thereby avoiding the mismatches observed in assay N°1. In the same way, samples from *S. salar* showed slighter differences in Ct values between assays.

**Table 2:** Comparative performance of the assays in different species of salmonid tested

	**ELF1 α**		**ELF1α GIM-1**		**ELF1α GIM-2**	
	**Ct value**	**ΔRn**	**Ct value**	**ΔRn**	**Ct value**	**ΔRn**
***O. mykiss***	32.31 ± 0.88	0.201 ± 0.039	26.49 ± 0.25	0.811 ± 0.019	21.43 ± 0.080	1.498 ± 0.017
***O. kisutch***	31.10 ± 0.36	0.238 ± 0.021	27.86 ± 0.17	0.686 ± 0.043	22.71 ± 0.109	1.353 ± 0.019
***S. salar***	28.55 ± 0.43	0.307 ± 0.021	29.18 ± 0,14	0.519 ± 0.008	23.56 ± 0.017	1.232 ± 0.001

The comparison was made based on the Ct values and relative fluorescence intensities (ΔRn). The probes used in this comparison were depleted of either MGB or LNA. The data are shown as a Ct value ± standard deviation and ΔRn ± standard deviation (n = 3).

Although both alternative assays (N°2 and N°3) considered the existing nucleotide variability of the species tested, the performance of assay N°3 was notoriously better than that of assay N°2. We hypothesize that this effect may be a result of a structural interference between the primer and the probe and not primarily of the template mismatches.

The next step in the evaluation was to consider the design of a more stable assay by increasing the Tm of the probes and thus increasing the annealing stability between the probe and the template. To achieve this objective, MGB was used on the probe of the ELF1α assay, as reported previously [13] and Locked Nucleic Acid (LNA) was used on the probe of the ELF1α GIM-2 assay. The comparison was made between assay N°3 (selected new assay) and the original assay N°1 (reference assay) considering samples derived from both tissues and cell cultures from different species of salmonids. Table 3 summarizes our results, showing that, as expected, cell culture and tissues samples from *S. salar* had lower ΔCt values between assays (Ct value ELF1α - Ct value ELF1α GIM-2), while cell culture and tissues samples from the *Oncorhynchus* genus had higher differences in Ct values between assays and therefore a higher ΔCt. The largest differences, however, were observed between samples derived from *O. tshawytscha,* where a maximal ΔCt value of 11.101 was obtained from tissue-derived samples.

**Table 3:** Comparison of differences in Ct values in tissue and cell culture samples from different salmonid species

**Samples**	**Type**	**Specie**	**ELF1α**	**ELF1α GIM-2**	**Δ Ct value**	**Eficiency (%)**
			**Ct Value**	**Ct Value**		
ASK-1	*Cell culture*	*S. salar*	28.134 ± 0.149	25.381 ± 0.114	2.753	96.65
SHK-1	*Cell culture*	*S. salar*	27.198 ± 0.204	24.237 ± 0.078	2.961	92.78
RTS11	*Cell culture*	*O. mykiss*	30.384 ± 0.338	25.028 ± 0.191	5.356	96.52
CHSE-214	*Cell culture*	*O. tshawytscha*	32.768 ± 0.421	24.354 ± 0.262	8.414	93.64
GIMCP	*Tissue*	*S. salar*	29.235 ± 0.107	25.297 ± 0.205	3.938	96.90
GIM017	*Tissue*	*O. mykiss*	33.467 ± 0.63	26.194 ± 0.199	5.273	96.16
GIM025	*Tissue*	*O. kisutch*	29.447 ± 0.177	24.693 ± 0.146	4.754	101.24
GIM496	*Tissue*	*O. tshawytscha*	36.494 ± 0.217	25.393 ± 0.161	11.101	97.22

The ΔCt value was calculated (Ct value ELF1α - Ct value ELF1α GIM-2).

An additional analysis can be performed when we compare the results from Table 2 (probes without the enhancer Tm system) with the results from Table 3 (probes with the enhancer Tm system). In fact, differences between assays N°1 and N°3, shown in Table 2, gave a ΔCt value of 10.88 for the *O. mykiss* tissue sample, whereas in Table 3, the ΔCt value was diminished to 5.27. This is a powerful demonstration that the enhancers, in addition to increasing Tm values, stabilize the probe-template annealing and, as a result, render the assay more robust.

Although only LNA was used in the probe of the ELF1α GIM-2 assay, we would expect a similar result when using MGB as an enhancer.

The evaluation of the efficiency of assay N°3 for all samples tested is shown in Table 3. All values for the RT-qPCR reactions fell within the suitable experimental range (90%-110%) [27].

### Evaluation of field samples

The final evaluation compared assay N°3 (ELF1α GIM-2) with the original assay N°1 (ELF1α), using the same distribution of LNA as a system to increase the probe Tm value in both assays. Field samples from *S. Salar* (n = 98), *O. mykiss* (n = 97) and *O. kisutch* (n = 82) were obtained from different farms located in the south of Chile, and parallel assays were run under the same RT-qPCR conditions (reagent mix, software setup, and data analysis). Four (out of 82) samples from *O. kisutch* were withdrawn from the analysis because they could interfere with the correct analysis because they displayed ΔCt values over 9, and one of them had a ΔCt value of 16. This result suggested that this might be due to several mismatches between the template and the primers and the probe of the ELF1α assay.

**Figure 2:**
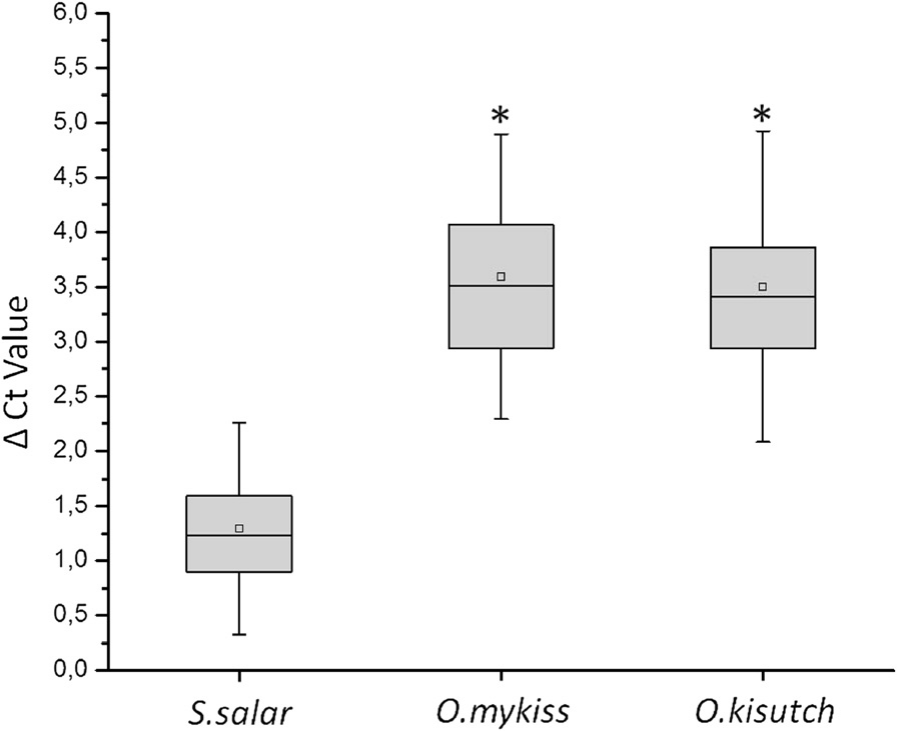
**ΔCt values from the ELF1α GIM-2 and ELF1α assays in field samples.** The ΔCt value was calculated (Ct value ELF1α - Ct value ELF1α GIM-2). In the plot, error bars are standard deviations from the average of all data. *S. salar* is significantly different from the *O. kisutch* and *O. mykiss* species, according to the one-way ANOVA following by a Tukey’s test (p < 0.05).

An integral analysis of the data shows that, similar to previous results, *S. salar* samples displayed smaller differences between assays N°3 and N°1, which was expected, as the latter was designed to specifically fit S*. salar* sequences. Samples from *O. mykiss* and *O. kisutch* displayed higher differences in Ct values, as seen in Figure 2. Therefore, ΔCt values depend on the salmonid species analyzed. Furthermore, no significant differences in ΔCt values were observed between the *O. mykiss* and *O. kisutch* samples analyzed.

**Figure 3:**
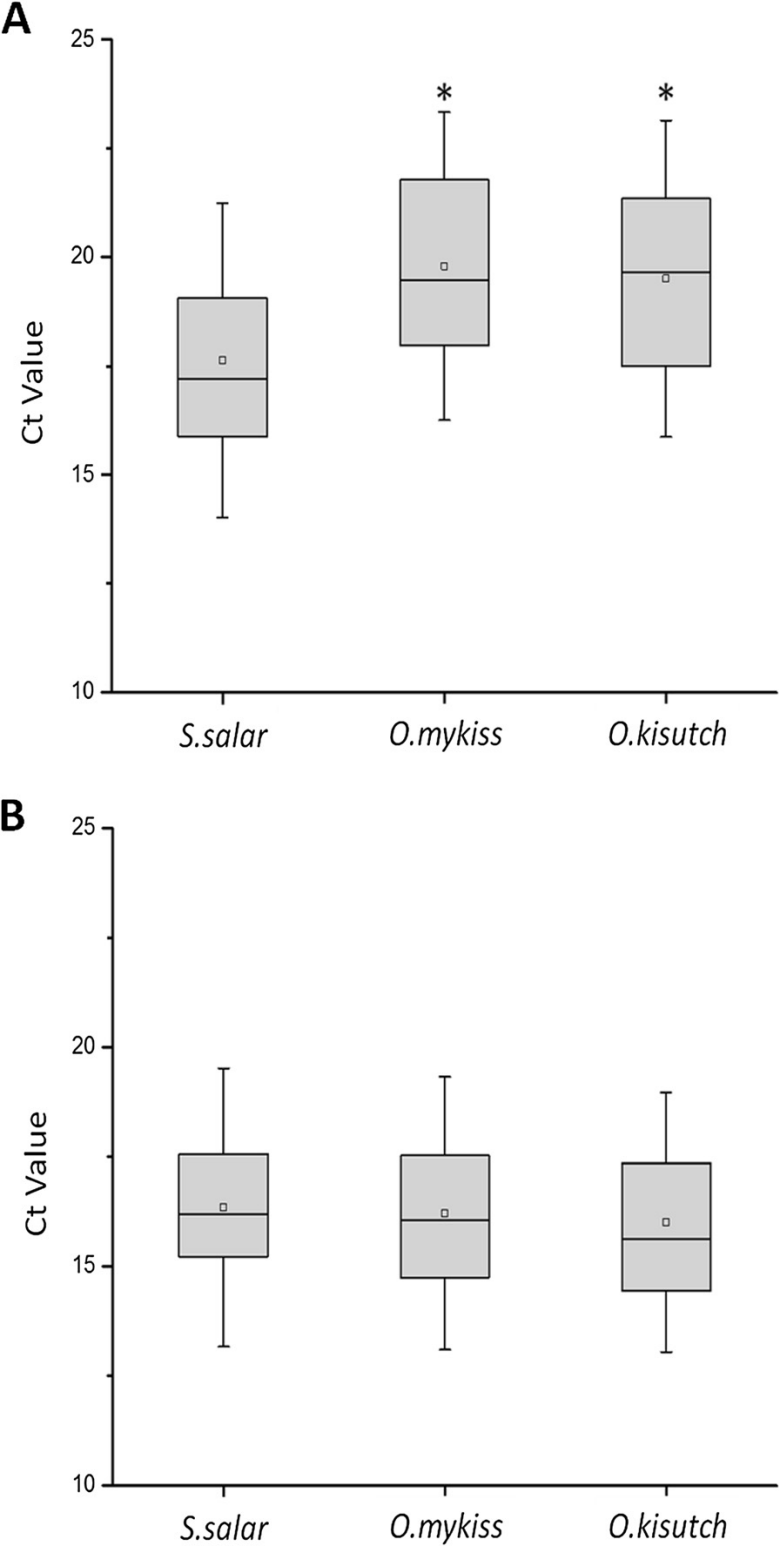
**Distribution of Ct values of field samples.** ELF1α assay **(A)** and the ELF1α GIM-2 assay **(B)**. The ELF1α GIM-2 assay does not show significant differences between the three salmonid species, whereas the ELF1α assay shows a significant difference between the *S. salar* and *Oncorhynchus* species samples (ӿ). Statistical analysis was performed using one-way ANOVA and Tukey’s test (p < 0.05).

Figure 3 shows the Ct values displayed in each assay from field samples of the three salmonid species. As expected, the new assay N°3 (ELF1α GIM-2) displayed no significant differences among the Ct values obtained (Figure 3B). When the original assay N°1 (ELF1α) was used, significant differences were observed between Ct values of samples from the *S. salar* and *Oncorhynchus* species (Figure 3A)*.*

These differences in Ct values reveal the importance in diagnoses of an adequate endogenous control. Indeed, using assay N°1, samples derived from *S. salar* display Ct values within a valid range for diagnostic purposes, whereas samples from the *Oncorhynchus* group display higher Ct values, some of which were beyond the valid range for diagnoses. Thus, incorrect diagnoses may occur as a result of a weak detection assay design; however, this effect is corrected in the new assay.

## Conclusions

Here we identified nucleotide variability in the amplified region of ELF1α, which causes highly variable results when the ELF1α assay is used as an endogenous control for *Oncorhynchus* species. Using this information, we have designed and improved a single reliable and efficient assay (ELF1α GIM-2), driven to an endogenous cellular quality control, to be used in RT-qPCR diagnostics of pathogenic agents in tissue samples from the salmonid species analyzed in this work.

## Methods

### Samples

Tissue samples analyzed were obtained from the kidneys of *Salmo salar, Oncorhynchus mykiss, Oncorhynchus kisutch* and *Oncorhynchus tshawytscha* according to official procedures established by Sernapesca, the Chilean government institution in charge of animal health surveillance in aquaculture and fisheries. Salmonid cell lines ASK-1, Atlantic Salmon Kidney (ATCC; CRL-2747) [28], SHK-1, Atlantic Salmon Head Kidney [29], CHSE-214, Chinook Salmon Embryo (ATCC; CRL-1681) [30] and RTS11, rainbow trout monocytes/macrophages cell line [31], were used as verification controls.

### Ethical statement

Tissue samples from fish were obtained from the surveillance program for fish disease in Chile. Fish were not killed for the purpose of this study. All sampling was performed according the regulations of Sernapesca (Chilean government institution in charge of fish health) and carried out in strict compliance with the recommendations of Chapter 7.4 of the Aquatic Animal Health Code of the World Organization for Animal Health. Every effort was made to minimize animal suffering in all procedures.

### Total RNA isolation

Tissue samples were preserved in RNAlater (Ambion) to be transported from the fish farms to the laboratory. Total RNA from the tissue samples and cell culture samples were isolated using the RNeasy Mini Kit (Qiagen), according to the standard protocols recommended by the supplier. Prior to the isolation of RNA, tissue samples were homogenized using a MagnaLyser device (Roche) for 30 sec at 6500 rpm. RNA samples were stored at -80°C until use.

### cDNA synthesis

RNA samples for sequencing were reverse transcribed using the SuperScript III kit (Invitrogen), according to the protocols recommended by the supplier. Briefly, approximately 1 μg of total RNA was added to the mix, which contained 0.5 μL of dNTPs (10 mM), 2 μL of 5 X First Strand Buffer, 0.5 μL of DTT (0.1 M), 0.5 μL of SuperScript III reverse transcriptase enzyme, and 1 μL of Random Primers (100 μM) (Fermentas) in a final volume of 10 μL. Reactions were incubated at 25°C for 10 min, 50°C for 1 h, and 75°C for 15 min. The final cDNA was diluted 3-fold before being used as a template in PCR.

### PCR and sequencing analyses

All PCR reactions for sequencing purposes were performed using external primers ELF1α Ext For 5′-ATG GGC TGG TTC AAG GGA TG-3′ and ELF1α Ext Rev 5′-CGT GGT GCA TCT CCA CAG AC-3′. PCR was performed using the Go Taq Flexi Polymerase Kit (Promega). Each reaction had a final volume of 25 μL and consisted of 5 μL of 5X Colorless Flexi Buffer, 0.5 μL of dNTP (10 mM), 3 μL of MgCL_2_ (25 mM), 0.2 μL of Go Taq DNA Polymerase, each primer at a final concentration of 400 nM, 11.3 μL of nuclease-free water (Invitrogen), and 4 μL of the diluted cDNA. The PCR program used consisted of an initial denaturation for 2 min at 95°C, followed by 40 cycles of denaturation for 30 sec at 94°C, primer annealing for 30 sec at 60°C, and elongation for 1 min at 72°C. The final elongation step was run for 5 min at 72°C. The resulting amplicons were resolved by agarose gel electrophoresis, and the target bands were purified from the gel using the E.Z.N.A Gel Extraction Kit (Omega Biotek), according to the protocols recommended by the supplier. The purified PCR products were quantified and then sequenced at Macrogen Inc. (Korea) with the same primers used in the PCR reaction. Sequences were interpreted using the Bioedit Software [24] and the multiple alignments were performed via ClustalW [23].

### Real-time RT-qPCR

Real-time PCR amplifications were performed using the Super Script III Platinum One-Step Quantitative RT-PCR System Kit (Invitrogen) and the StepOnePlus Real-time PCR System thermocycler (Applied Biosystems). Primer sets and assay names are listed in Table 1. Each reaction was carried out in a final volume of 20 μL. Primers were at a final concentration of 1 μM, and hydrolysis probes were at a final concentration of 0.3 μM. The reactions contained the passive reference dye, ROX. One cycle of reverse transcription was run for 15 min at 50°C, 1 cycle of denaturation for 2 min at 95°C, and 45 cycles of denaturation for 15 sec at 95°C, followed by annealing and elongation for 1 min at 60°C. Real-time PCR efficiencies were calculated from the slope according to the established equation E = 10 ^(-1/slope)^[32].

### Field sample evaluation

Samples consisted of organ pools (heart, gill and kidney) from 98 specimens of S. *salar*, 97 of O. *kisutch* and 82 of O. *mykiss.* Samples were fixed with RNAlater in a tissue:fixator ratio of 1:10 and stored at 6-8°C prior to analysis. The fixed samples were homogenized using Precellys device (Bertin Technologies). The homogenized samples were centrifuged by 2 min. at 13.000 r.p.m (pico 17, Thermo). The supernatants were purified for RNA using a high purity viral nucleic acid kit (Roche) according to the manufacturer’s instructions. The purified RNA was analyzed using the RT-qPCR protocols described previously.

### Statistical analysis

The statistical analyses were performed using OriginPro V8.5 software. Comparisons between Ct values or ΔCt values were performed using one way ANOVA followed by a Tukey’s tests (P < 0.05), to denote significant differences.

## Abbreviations

ELF1α: Elongation factor 1 alpha; PCR: Polymerase chain reaction; RT-qPCR: Real-time reverse transcription polymerase chain reaction.

## Competing interests

The authors declare that they have no competing interests.

## Authors’ contributions

DS and SM designed the experiments, performed data analysis and drafted the manuscript. HB and HG were in charge of the field samples in the experimental part. AL was in charge of the sampling process and designed the experiments. All authors have read and approved the final manuscript.
